# Surgeon-General of New York

**Published:** 1865-02

**Authors:** 


Surgeon-General of New York.—Dr. S. D. Willard, of Albany, has been appointed Surgeon-General of the State of New York on the staff of Gov. Fenton. A more efficient and capable officer could not have been selected.

---

The eminent surgeon, M. Nelaton, has been honored with a costly and handsome gold medal, from the Italian residents of Peru, as an expression of their appreciation of the eminent services rendered by him to Garibaldi.

